# Experimental Investigation of the Effect of Adding Nanoparticles to Polymer Flooding in Water-Wet Micromodels

**DOI:** 10.3390/nano10081489

**Published:** 2020-07-29

**Authors:** Edgar Rueda, Salem Akarri, Ole Torsæter, Rosangela B.Z.L. Moreno

**Affiliations:** 1 School of Mechanical Engineering, University of Campinas, Rua Mendeleyev, 200 Cidade Universitária Barão Geraldo, Campinas–SP CEP 13083-860, Brazil; zanoni@fem.unicamp.br; 2 PoreLab Research Centre, Department of Geoscience and Petroleum, Norwegian University of Science and Technology (NTNU), S. P. Andersens veg 15a, 7031 Trondheim, Norway; ole.torsater@ntnu.no

**Keywords:** enhanced oil recovery, chemical flooding, biopolymer, silica nanoparticles, microfluidics

## Abstract

Recently, the combination of conventional chemical methods for enhanced oil recovery (EOR) and nanotechnology has received lots of attention. This experimental study explores the dynamic changes in the oil configuration due to the addition of nanoparticles (NPs) to biopolymer flooding. The tests were performed in water-wet micromodels using Xanthan Gum and Scleroglucan, and silica-based NPs in a secondary mode. The microfluidic setup was integrated with a microscope to capture the micro-scale fluid configurations. The change in saturation, connectivity, and cluster size distributions of the non-wetting phase was evaluated by means of image analysis. The biopolymer content did not affect the ability of the NPs to reduce the interfacial tension. The experiments showed that the reference nanofluid (NF) flood led to the highest ultimate oil recovery, compared to the Xanthan Gum, Scleroglucan and brine flooding at the same capillary number. In the cases of adding NPs to the biopolymer solutions, NPs-assisted Xanthan flooding achieved the highest ultimate oil recovery. This behavior was also evident at a higher capillary number. The overall finding suggests a more homogenous dispersion of the NPs in the solution and a reduction in the polymer adsorption in the Xanthan Gum/NPs solution, which explains the improvement in the sweep efficiency and recovery factor.

## 1. Introduction

One of the most common enhanced oil recovery (EOR) techniques is polymer flooding. That is described by adding polymer molecules to the aqueous injected phase aiming to increase the viscosity of the solution to be injected, reducing the mobility ratio of water to oil and to controlling the fingering effects. However, polymer waterfloods are highly probable to result in permeability reduction [1]. In addition, polymer flooding may be considered as an improved waterflooding method rather than an EOR method, since it does not ordinarily unlock the isolated residual oil by water in the porous medium [2]. The efficiency of a polymer flooding is related to three aspects: (1) the reduction in the injected fluid mobility, (2) polymer alteration of fractional flow curves, and (3) diversion of the injected water from swept areas [3].

The Xanthan Gum and Scleroglucan are two types of microbial-origin biopolymers. The first polymer is obtained from the microorganism Xanthomas Campestris [4], and the second is a class of fungal polysaccharides secreted extracellularly by fungi of the genus Sclerotium [5]. Xanthan Gum is an anionic polymer with an estimated molecular weight of 2.65 Million of Daltons, the Pyruvate and Acetate content are 0.9%, and 3.52%, respectively [6]. The Scleroglucan is a neutral β-1, 3-β-1,6-glucan. X-ray diffraction shows a triple-helical conformation in the solid-state [7]. In aqueous solution, the Scleroglucan molecule exists in a stiff, triple-stranded helical structure, where side chains are exposed toward the exterior [8]. The Scleroglucan molecular weight is approximately 5.2 Million of Daltons [9].

The biopolymers have been applied in the North Alma Penn unit [10]. In the early stages of the waterflooding and using concentrations from 250 ppm to 304 ppm, the results showed no damage in the formation, and the decline in the water production in some production wells could be attributed to the positive effect of the biopolymers. In the case of the field Eddesse-Nord sandstone in Germany [11], the implementation of a Xanthan Gum pilot project at 800 ppm of concentration showed successful injection results, in terms of lower values of adsorption than expected and low degradation effects.

Nanotechnology has received much attention in different engineering disciplines in the oil industry. An important example is the positive impact on the oil recovery factor because of the improvement in the sweep efficiency and reduction in the trapped oil by the capillary pressure [12,13,14]. The four mechanisms investigated in literature as oil displacement mechanisms by NPs are disjoining pressure gradient at oil–NPs interface, the density difference between water and NPs, wettability alteration, and reduction in interfacial tension [15]. The disjoining pressure is defined as the pressure required to oppose the fluid/solid attractive forces and lift the film from a solid surface. In other words, this pressure represents the net pressure difference between the pressure in a thin film and that in the bulk liquid from which the thin film extends [16]. The density difference between the nanoparticle and the water drives the particles to agglomerate in the smallest pores and throats. That difference generates an increase in the pressure, which mobilizes the oil in the adjacent pores [17]. NPs have the ability to alter the wettability from oil-wet to neutral or more water-wet surface [15]. The use of hydrophilic silica NPs has shown a reduction in the interfacial tension between the brine and oil when the concentration of NPs increases [18,19].

In the last decade, the combination of the polymer flooding technique and NPs has been investigated as a promising method to reduce trapping efficiency. NPs (silica NPs, surface-modified silica NPs, or nanoclay) have been added to polymer solutions (hydrolyzed polaycarylamide (HPAM) or Xanthan Gum (XG)) to improve the performance of polymer waterflooding. A micromodel-study presented that silica NPs and HPAM flooding increased the oil recovery by 10% of original oil in place (OOIP) than polymer flooding due to the wettability alteration mechanism [20]. Another micro-scale study [21], presented that the efficiency of the pore-to-pore displacement of heavy oil was improved by an increase in the concentration of silica NPs in polaycarylamide (PAM) solutions, even when salt was present in the system. That was explained by ion-dipole interactions between the NPs and cations reducing the polymer degradation. Their study shows that alteration is more effective at lower polymer concentrations. However, for field applications, the use of HPAM is more common than PAM due to dilution problems of PAM. Cheraghian and Khalilinezhad, 2015 [22] tested the addition of nanoclay (at three concentrations: 0.8, 0.9, and 1.0 wt.%) to polymer flooding (at different concentrations of HPAM) on core-scale. They found that the best recipe resulted in incremental heavy oil recovery by a factor of 5% of OOIP in comparison to polymer flooding after one pore volume fluid injection. Another experimental work showed that silica NPs and nanoclay increased the PAM solution viscosity and decreased its adsorption onto the rock [23]. The core-scale work by Saha et al., 2018 [24] showed that nanoparticle assisted polymer (Xanthan Gum) flooding was effective for EOR applications in heavy crude oil systems. Based on the stability analysis of silica NPs, the NPs were more stable in the polymer solution than in water. Besides, the viscosity of the polymer solution improved, and the interfacial tension reduced after the addition of NPs. A considerable improvement in sweep efficiency was observed by adding surface-modified silica NPs to XG solutions, but not to HPAM solutions [25]. Mohammadi et al., 2019 [26] studied the effect in the rheology of polymeric solutions with silica NPs. Their results showed that the viscosity of PAM solutions increased when the concentration of NPs was respectively 0.5, 1.0, 1.5, and 2.0 wt.%. A faster increase in thickness occurred when the concentration was higher than 1.5 wt.% at 25 °C. This point corresponds to the critical nanoparticle concentration (CNC). Kennedy et al., 2015 [27] found in a rheological study that Xanthan Gum exhibited shear-thinning over the entire shear rate range and enhanced the viscosity and the storage moduli with the increasing particle concentration. This phenomenon created larger domains of the associated polymer gel as a result of the interaction between the polymer and the particles.

Microfluidics has become a vital research area in the petroleum industry. It is highly appreciated owing to significantly improving our current understanding of pore-scale displacement events and interactions occurring within tiny fluid volumes, moving within well-defined pore-structures. In addition, it enables capturing the dynamic changes within the medium with a high spatial and temporal resolution. The energy and chemicals fed to a microfluidic setup are much less compared to the core-flooding apparatus, which lowers costs and risks. The heavy and conventional core flooding setup is reduced to a smaller apparatus with a higher level of accuracy and significant observations, more tests can be performed to validated and compare different flooding scenarios and the result takes part in the selection criteria previous to more complex tests like core-flooding.

The ratio of the viscous forces to the capillary forces defined as the capillary number (*Nc*), see Equation (1), indicates what forces dominate the flow regime, viscous or capillary flow. Where *V_w_* is the interstitial velocity, *µ* is the viscosity and *σ_ow_* is the interfacial tension between the fluids involved in the displacement. In conventional brine flooding, the increase in the injection rate (higher capillary number) can reduce the trapped oil in the reservoirs and glass-bead packs [28,29]. In the case of fluids with NPs, the tendency is different because the NPs require time to modify the wettability of the surface by disjoining pressure [29] or because at higher rates the particles agglomerate in the porous media [13,29]:

(1) $$N_{c}=\frac{V_{w}{\;\mu }_{w}}{\sigma_{ow}}.$$

The water injectivity (*I_w_*) during a water flooding process through a porous media is proposed by Civan, 2016 [30] in Equation (2) where *q_w_* is the water rate and *ΔP* is the pressure difference across the porous medium:

(2) $$I_{w\;}=\frac{q_{w}}{∆P}.$$

The injectivity index was used in this work to evaluate the effect of each solution during the displacement, according to a reference solution (brine in the case of the fluids without NPs and NPs in the case of the solution with silica NPs). That is calculated using Equation (3):

(3) $$I_{index}=\frac{I_{polymer}}{I_{reference}}.$$

Modeling these phenomena is challenging. Simulation models need to take into consideration a multi-phase multi-component interactive system. It is necessary to represent the effects of the nanoparticles on the interfacial tension, capillary pressure, and wettability. Polymer flooding also includes interactive phenomena. The non-Newtonian behavior of the polymer solutions requires local and instantaneous determination of the displacing fluid viscosity as a function of shear rate and polymer concentration. However, retention and degradation phenomena strip polymer from the solution, which justifies the multicomponent approach. Changes in local permeability can take place because of polymer adsorption. All those phenomena have recently received close attention, and semi-analytical procedures [31] or commercial simulators [32] are progressively coupling their effects on chemical enhanced oil recovery. Nevertheless, the associative contributions of nanoparticles and polymer is new, opening opportunities for developments in this area.

In this work, we study the improvement in the sweep and the displacement efficiencies by the polymer flooding using Xanthan Gum and Scleroglucan assisted with silica NPs. Both phenomena were evaluated independently and simultaneously from two-dimensional (2D) image analysis obtained during microfluidics tests in water-wet glass micromodels and translated in terms of oil recovery factor and injectivity loss. The combination of both chemicals represents an environmentally friendly alternative to improve conventional water flooding. This study also provides new insight into the application of NPs in the oil and gas industry

## 2. Materials and Methods

### 2.1. Biopolymers, NPs, and Brine

Two different types of dry based biopolymers; Xanthan Gum and Scleroglucan were diluted in deionized water to reach a concentration of 0.4 wt.%. To have full hydration of the polymers, they were mixed using a magnetic stirrer for 24 h for the Xanthan Gum and seven days for the Scleroglucan at room temperature. Those solutions are the stock to prepare the dilutions.

The silica nanoparticle used in this study is a special laboratory research development for Evonic. The name of AEROSIL^®^ originally markets the product. The main component was silicon dioxide with other minor parts of alumina. The provided particles have attached chains of polymers to their surfaces to ensure long-term stability in solution. This type of nanomaterial is known as polymer-coated silica nanoparticles. The solution in gel was supplied as by the name AERODISP^®^. The charge of this type of nanoparticle is anionic equal to the Xanthan Gum, while Scleroglucan molecule is neutral. The properties of the NPs are given in Table 1. The 3 wt.% sodium chloride brine was prepared using a magnetic stirrer for 30 min in deionized water. The density was 1.019 g/cm^3^, the viscosity of the solution was 1.04 mPa·s and the pH is 6.52. All the values were measured at room temperature (22 °C).

Three groups of dilutions were prepared from the stock solutions. The first group is composed of the brine and the 0.1 wt.% NPs solution. In the second group, two polymeric solutions were prepared using biopolymers, Xanthan Gum or Scleroglucan, and in the last group, at each polymeric solution, NPs were added. Overall, six solutions were prepared in the concentrations shown in Table 2. Each solution was mixed for 30 min using a magnetic stirrer at 500 rpm and the solutions were stored at 4 °C.

### 2.2. Crude Oil

Dead oil from the North Sea was used in this study. The filtration process was performed using a filter whose pore size was 1.1 µm. The density and viscosity measured at 22 °C were 0.892 g/cm^3^ (27 °API), and 21.9 mPa·s, respectively. Saturate, aromatic, resin and asphaltenes (SARA) analysis showed that the oil contains 71.57% of saturates, 20.81% of aromatics, 7.44% of resins and 0.18% of asphaltenes.

### 2.3. Glass Micromodel

**Figure 1:**
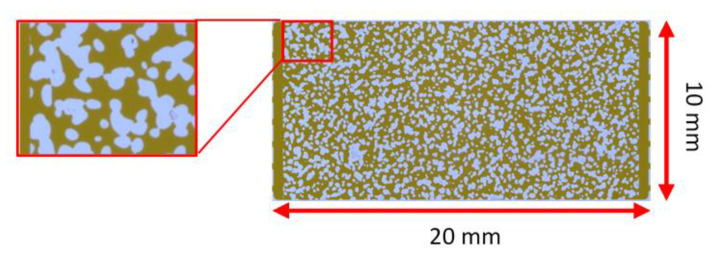
Pore-network of the glass micromodel used in this study (Blue: grain area; brown: pore area).

Borosilicate glass micromodels chips were used in the study (Figure 1). Those models represent a real porous media network, and under a microscope, due to the clear optical properties from all sides, allowing the understanding of the flow dynamic behavior of the wetting and non-wetting phases in a displacement process. The size of the chip was 45 mm × 15 mm and the thickness was 1800 µm. The dimensions of the pore-network were 20 mm × 10 mm and the thickness was 20 µm. According to the manufacturer specifications for the chip, the pore volume was 2.3 µL, the porosity was 57% and the permeability was 2.5 Darcy. The porosity value was also confirmed by the image analysis. Single-phase flooding tests were performed to estimate the liquid permeability of the pore-network according to the work presented by Pradhan et al., 2019 [33]. The network permeability was around 8.3 Darcy.

### 2.4. Micromodel Setup

**Figure 2:**
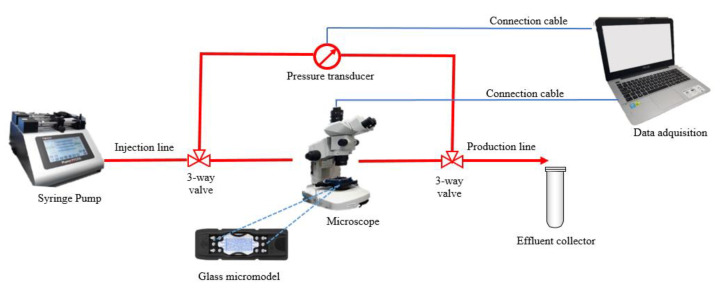
Diagram of the micromodel flooding setup.

The micromodel was attached to an aluminum platform of 128 mm × 85.4 mm × 20 mm that operates at a maximum of 10 bar of pressure and 80 °C. The inlet and outlet ports are connected using Teflon tubing. The visualization was done under a microscope equipped with a digital camera used to capture images every 30 s. The fluids were injected using a syringe pump. A pressure transducer was adapted in the inlet and outlet lines to record the pressure during the recovery agent injection. Figure 2 shows a representation of the micromodel set up.

### 2.5. Interfacial Tension and Contact Angle

The interfacial tension test was made using a drop shape analyzer and using a J-shaped needle with a diameter of 1.001 mm. The fluid container dimensions are 30.0 mm length, 20.0 mm width and 30.6 mm height, at room conditions. The pendant drop method was used to analyze the drop shape of oil against the aqueous phase and with the Young-Laplace equation (Equation (4)) to calculate the interfacial tension.

(4) $$\sigma =\frac{{\Delta \rho \cdot g\;\cdot R}_{o}}{\beta }$$

where:

*σ* = interfacial tension.
*g* = gravitational constant.
*R_o_* = radius of drop curvature.
*β* = shape factor.
Δ*ρ* = density difference

The measurement was performed for all solutions for 2 h to reach the equilibrium condition and it was repeated to confirm the result.

The contact angle between a drop of crude oil and the polymer solutions was measured using the same apparatus. The solid surface used was glass and the oil drop was placed under the surface (captive bubble method).

### 2.6. Nanosize Distribution

The particle size distribution is measured using a Dynamic Light Scattering (DLS) instrument. The apparatus is a Zetasizer Nano series, where the diameter of the sphere that diffuses in the solution is measured from the Brownian motion of the particles in a sample. DLS and established theories were used to determine the particular size distribution and the relationship with the diffusion speed.

### 2.7. Microfluidics Tests

All tests were developed using a secondary methodology to evaluate the effect of different recovery agents in terms of oil recovery factor, pressure drop and sweep efficiency. The chips were cleaned using toluene, methanol, acetone, and distilled water and then dried in an oven at 60 °C overnight. The brine saturation was performed under vacuum (~80 mTorr). The brine was injected at different rates to push out any remaining air in the chip.

**Figure 3:**
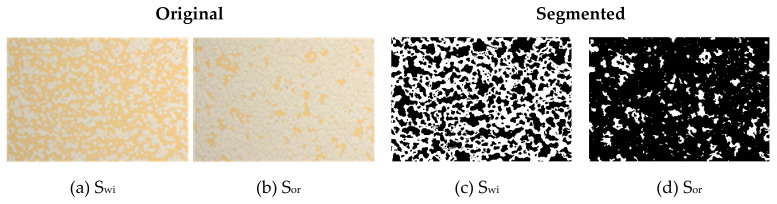
Original and segmented images of the pre- and post-flooding state in the microchip: (**a**) initial water saturation—original; (**b**) residual oil saturation—original; (**c**) initial water saturation—segmented; (**d**) residual oil saturation—segmented.

The oil injection was performed at 1, 10, 20, 30, 70 and 100 µL/min until no more water was produced. Under this condition, the remaining brine in the chip is the connate water and the oil saturation is the maximum. The microfluidic tests were divided into three parts. The first four tests were performed at a capillary number of 1 × 10^−6^ in order to evaluate the biopolymer solutions in comparison with the brine at the same flow regime. In the second part, two more experiments were developed at the 2 × 10^−6^ capillary number. In this case, Xanthan Gum solutions assisted with the NPs and the reference nanofluid (NF) were studied. Finally, the next two tests evaluated the reference NF and the Xanthan Gum solution with NPs at a higher capillary number by increasing the flow rate to 0.39 µL/min to see the effect of a high rate injection on the performance of the solutions. The recovery agent was injected until no more oil was produced. Due to the differences in the performance of each solution, different amounts of fluid are necessary to reach steady-state conditions in each test. The dynamic study was made taking images every 30 s during the recovery agent injection and the automatic image analysis was done using a Matlab routine. The time was reported as porous volumes injected (PVI). This was calculated from the porous volume of the entire chip. However, the image analysis was performed in a representative area located in the center of the chip, which is 40% of the total area. The segmentation uses the color thresholding method to extract the oleic phase. That results in binary images were analyzed to evaluate the total area of oil and the number, size, and distribution of the clusters as the function of the time and the characteristic Euler number. According to [34], for a 2D image, the Euler number is defined as the number of connected components—the number of holes. Figure 3a,b show the initial condition Swi (initial water saturation) and the final condition Sor (residual oil saturation) from the original images of the micromodel, where the oleic phase corresponds to the yellow part. The images after the treatment are shown in Figure 3c,d, where the white area corresponds to the oleic phase and in black refers to the porous media, brine and recovery agent.

## 3. Results

### 3.1. Fluids Characterization

The use of polymeric solutions has the objective of increasing the viscosity of the solution to be injected to reduce the mobility ratio between the oil and the injected fluid. In this study, the biopolymers increased the viscosity of the brine more than two times. A little additional increase in thickness was reached when the NPs were added, except for the Xanthan Gum where the viscosity reduced by 2.65%. The NPs increased the pH of the solution on an average of 6.21%. Density measurements were based on the oscillation U-tube method. No considerable changes in the results of density measurements of the aqueous solutions were observed after adding NPs. The measured values of the viscosity and the density of the filtered crude oil sample from the North Sea (Dead Oil) were 21.9 mPa·s, and 0.892 g/cm^3^, respectively. The results are shown in Table 3.

### 3.2. Nano-Size Distribution

According to the supplier of the silica-NPs, the average size of the NPs is 32 nm. Even so, measurement of nano-size distribution was conducted to confirm the distribution of the NPs into the solutions (Table 3). The results showed 64.82 nm for the NF solution without biopolymers. This value is higher than that reported by the supplier because some particles could be agglomerated. That difference on the agglomeration is not significate, but besides that, the measurements were performed under static conditions. Therefore, agglomeration with higher nano-size distribution could happen under dynamic flow conditions through the porous media. This behavior is probably controlled when the biopolymers is added in the solution, which showed values for the average size of 31.87 nm for XG_NP and 31.48 nm for SCL_NP. This result could indicate a better distribution of the NPs when the biopolymers are in the solutions.

### 3.3. Interfacial Tension and Contact Angle

**Figure 4:**
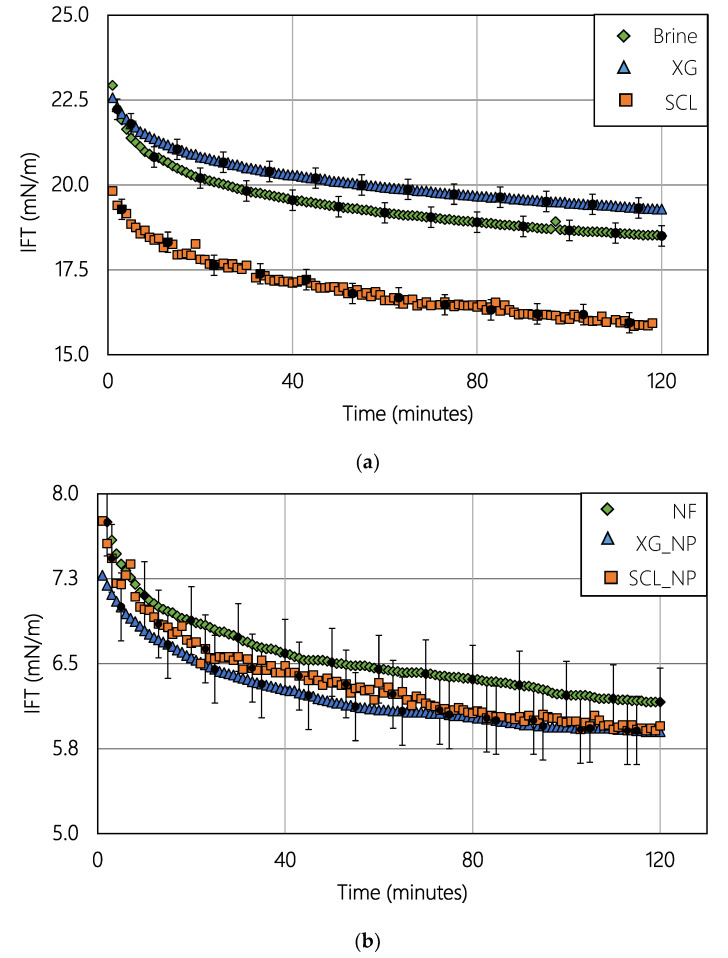
(**a**) Interfacial tension-free of NPs and (**b**) Interfacial tension in solutions with NPs.

The addition of hydrophilic silica NPs into the brine-oil system reduced the interfacial tension (IFT). Comparing the brine (Figure 4a) with the NF solution (Figure 4b), the IFT of the oil-NPs solution is one-third of the oil-brine system. When the biopolymer is present in the solution, the interaction between the polymers and the NPs did not affect the reduction due to NPs. The polymers slightly improved the reduction effect in the interfacial tension.

**Figure 5:**
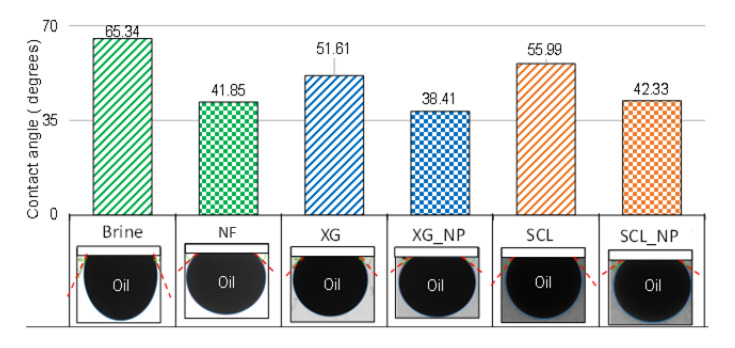
Results of the contact angle measurement for the fluids used in this study.

In the case of the contact angle, the behavior is similar to the IFT, where the NPs at 0.1 wt.% reduced the contact angle by 36% in comparison with the brine. However, when the biopolymers are in the solution, the reduction in the contact angle was less evident, showing values within 25% on average. Those results are shown in Figure 5.

### 3.4. Microfluidic Experiments

The screening process was made as a function of the type of fluids. For this reason, the flow regime was maintained constant based on the concept of capillary number. The porous media and the oil phase were the same for all tests. Secondary injection methodology and water-wet glass micromodels were used in the microfluidics tests. The initial water saturation was established by using a brine with 3 wt.% sodium chloride in all experiments. The same methodology was performed for brine and oil saturation to have the same initial condition previous to the recovery agent injection. The validation of the methodology was done performing arbitrarily the third test (SCL) more than one time. The results showed relative difference of 2.87% in the ultimate oil recovery factor at 3.4 PVI. That difference was considered as acceptable for the objective of the paper and was used as an argument to validate the microfluidic laboratory methodology used in this work.

#### 3.4.1. Microfluidic Screening of Biopolymer Solutions

In the first part of the study, three microfluidics tests were performed. The capillary number was constant at 1 × 10^−6^ to have the same flow regime in the porous medium. The flow rate for each test is shown in Table 4. In this case, brine with the biopolymers Xanthan Gum and Scleroglucan were evaluated.

**Figure 6:**
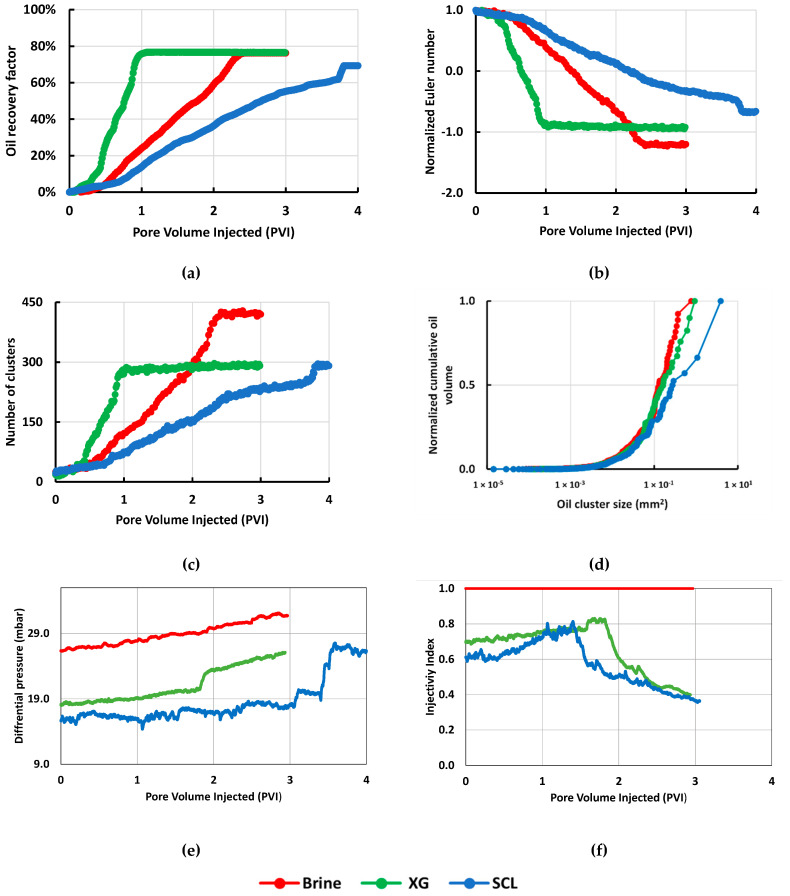
Microfluidic results for screening of biopolymer solutions at N_c_ = 1 × 10^−6^: (**a**) Oil recovery factor; (**b**) Normalized Euler number; (**c**) Number of clusters; (**d**) Normalized cumulative oil volume; (**e**) Differential pressure; (**f**) Injectivity index.

The biopolymers solutions as a recovery agent were studied and compared with conventional water flooding. According to Figure 6a, the Scleroglucan showed poor behavior obtaining the lowest oil recovery factor (69%) and the most delayed equilibrium point (3.8 PVI). The second biopolymer, Xanthan Gum, reached the same recovery as the brine (76%). However, the production with the biopolymer injection reaches the steady-state after the first porous volume injected, while the brine takes 2.4 PVI to reach the equilibrium. In terms of the number of oil clusters (Figure 6c) and oil connectivity (Figure 6b), the remaining number of clusters, the results for Xanthan Gum and Scleroglucan were the same, but the connectivity between those oil bodies was higher for the Scleroglucan. Finally, Figure 6d shows the relation between the number and size of the clusters. The tendency is similar for the brine and the Xanthan gum. In the case of the Scleroglucan, the graph indicates bigger remaining oil clusters after reaching the steady-state flow condition. Table A1 (Appendix A) shows the segmented images of the chip during the injection for each solution as a function of the porous volume injected (PVI).

The pressure response during the recovery agent injection is shown in Figure 6e. The results have a concordance with the flow rate, and in consequence, higher rates correspond to higher differential pressure. The hydrodynamic resistance during polymer flooding generate a pressure increase during the injection of the fluids without NPs. In terms of the injectivity index, Figure 6f shows a reduction in the injectivity when the biopolymers are injected in comparison with the brine injection.

#### 3.4.2. Effect of Adding NPs to Xanthan Gum Solutions

In the second part of the study, two more microfluidics tests were performed to evaluate the effect of the NPs and biopolymer as recovery agents. The capillary number was 2 × 10^−6^. The details for each test are shown in Table 5. NPs-assisted Scleroglucan flooding was not successful for the investigated application due to significant injectivity problems.

**Figure 7:**
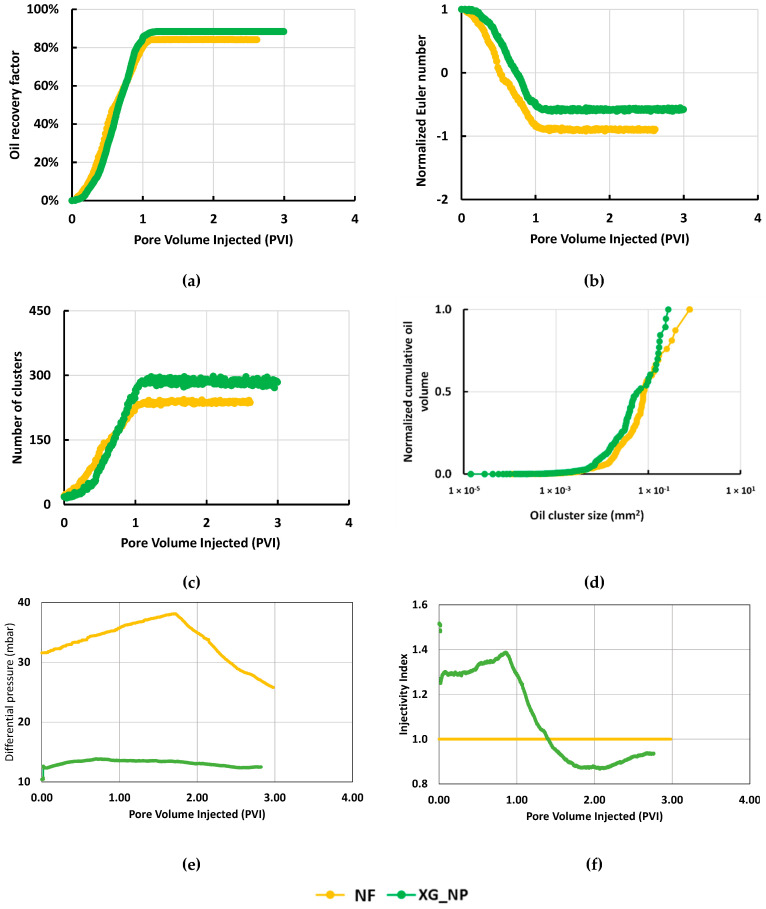
Microfluidic results showing the effect of adding NPs to xanthan gum solutions at N_c_ = 2 × 10^−6^: (**a**) Oil recovery factor; (**b**) Normalized Euler number; (**c**) Number of clusters; (**d**) Normalized cumulative oil volume; (**e**) Differential pressure; (**f**) Injectivity index.

The effect of the NPs in the Xanthan Gum solution was studied as a function of the oil recovery factor and the non-wetting clusters. Figure 7a showed an increase in the ultimate recovery from 84% for the NF solution to 88% for the Xanthan Gum solution with the silica NPs. For both fluids, the steady-state is reached after the injection of one pore volume. According to Figure 7b,c, the number of oil clusters and the connectivity was lower to the NF system compared to the biopolymer/NP system. However, Figure 7d showed that the size of the clusters is smaller for the solution with Xanthan Gum and for this reason, the recovery is higher. The segmented images used in the calculus are shown in Table A2 (Appendix A) as a function of the time (PVI) for each recovery agent.

In the case of differential pressure, hydrodynamic resistance generates a pressure increase. However, after the end of the oil production, the pressure decreases due to the difference in the fluid viscosity. Figure 7e shows that the ΔP for XG_NP decreased to a third compared to the NF fluid. The relation between the injection rate and the differential pressure in terms of the injectivity index (Figure 7f) shows an improvement in the injectivity of the system biopolymer-NPs in comparison with the solution without Xanthan Gum at N_c_ = 2 × 10^−6^.

#### 3.4.3. Effect of the Flow Rate on the Recovery Performance

In the third part of the study, two more microfluidic tests were performed at a high and constant rate to evaluate the effect of the biopolymer on the NPs behavior in an unfavorable flow regime scenery for the NPs. The properties of each test were shown in Table 6.

**Figure 8:**
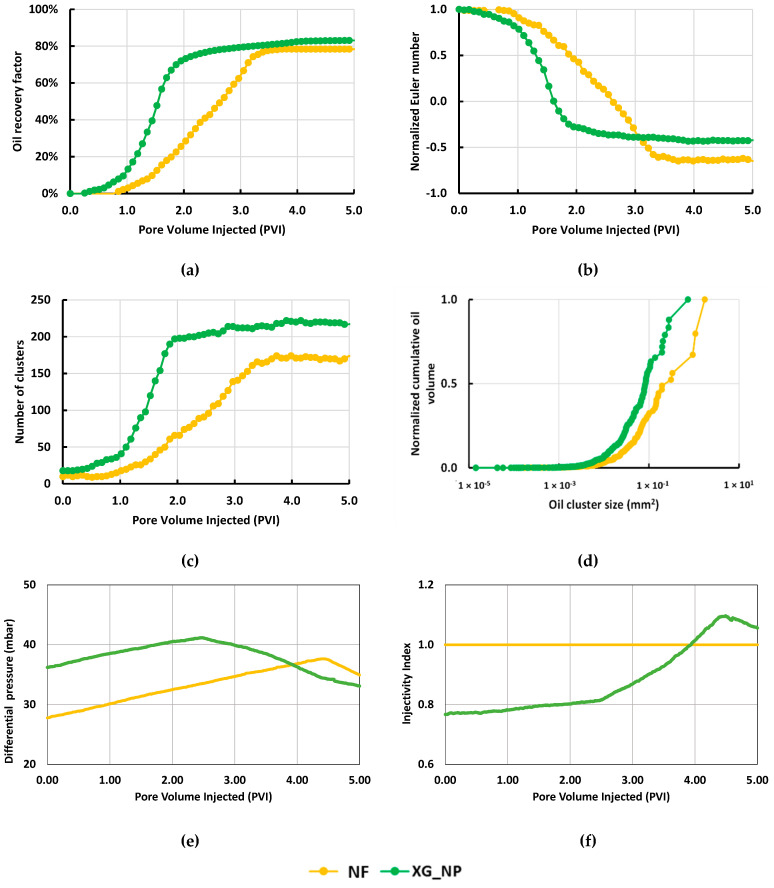
Microfluidic results showing the effect of a higher flow rate (0.39 µL/min) on the recovery performance: (**a**) Oil recovery factor; (**b**) Normalized Euler number; (**c**) Number of clusters; (**d**) Normalized cumulative oil volume; (**e**) Differential pressure; (**f**) Injectivity index.

In Figure 8a, the Xanthan Gum with the silica reached a higher recovery (85%) in comparison with the solution free of the biopolymer (78%). Additionally, the steady-state was reached faster for the biopolymer/NPs system. The study of the clusters in Figure 8b,c shows a higher number of clusters and higher connectivity for the XG_NP recovery agent. Nevertheless, according to Figure 8d, the size of the oil bodies was bigger in the NF flooding. In consequence, the biopolymer improved the sweep efficiency. Those graphs were built using the segmented images shown in Table A3 (Appendix A).

Finally, the pressure response is shown in Figure 8e. Higher values of differential pressure for the XG_NP due to the viscosity increase of the biopolymer solution are observed. According to Figure 8f, Xanthan Gum, at a high rate, loses injectivity relative to the NF solution during the first 4 PVI. Thereafter, the injectivity for the system with Xanthan Gum is better than the other fluid. The effect of the hydrodynamic resistance is similar to the previous test. However, the inflection point is delayed because of the end of the oil production was delayed too.

## 4. Discussion

### 4.1. Performance of the Xanthan Gum and Scleroglucan

The performance of the solutions used in this study was evaluated comparing the reference fluids (Brine and NF) and the biopolymer solutions with NPs. In this way, were tested the main properties of the polymers and NPs. The main objective of adding polymers to the brine was to increase the viscosity to improve the sweep efficiency of the water flooding. Table 7 showed an increase of more than two times the viscosity of the biopolymer solutions in comparison with the brine. Additionally, the interaction with the Silica NPs increased this measure in 0.1 mPa·s in the Scleroglucan and the reference fluid (NF). In the xanthan gum solutions, the NPs reduced the viscosity in 0.1 mPa·s. However, no evidence of relevant polymer degradation was shown when the NPs were added to the polymer solution.

The interfacial tension reduction was a property of the NPs solutions [18,19]. The results of Table 7 suggested a reduction of 13.5 mN/m for the solutions with Xanthan Gum, in consequence, the reduction was improved in 1.1 mN/m in comparison with the reference fluids (NF and brine). On the other hand, the reduction was 10.1 mN/m for the Scleroglucan. That reduction was lower than the value reached with the reference fluids (12.4 mN/m). The interaction solid-liquid was evaluated measuring the contact angle. The behavior of the biopolymer in the reduction was less evident than the reference fluids.

The stability of NPs solutions is a key point because the tendency of agglomeration precipitation. The measured of 64.8 nm (approximately two times the size reported by the manufacture) in the nanosize distribution for the reference NF solution is an indication of agglomeration. However, the results of this study suggested an improvement in the NPs stability and distribution due to the biopolymers presence in the solutions. That because the values obtained (Table 7) are very close to the real size of the particles (32 nm). The interaction between the NPs and the biopolymer generates a more alkaline solution according to the increase in the pH (Table 7).

### 4.2. Evaluation of the Recovery Factor

**Figure 9:**
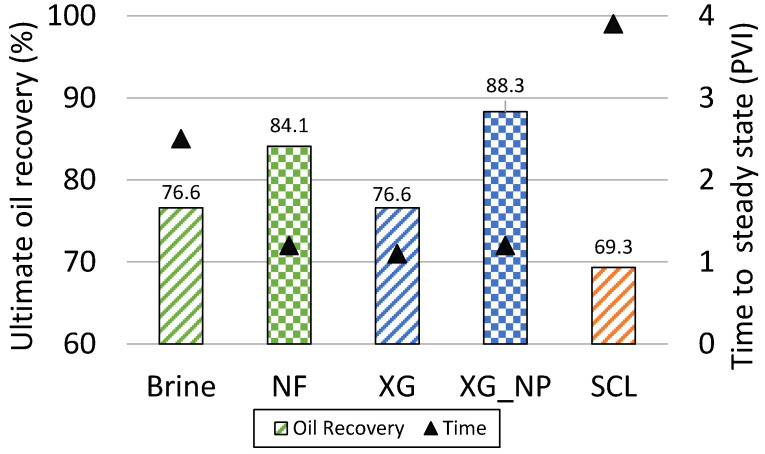
Ultimate oil recovery factor for the solutions evaluated using microfluidic tests as a function of the time to reach the steady-state.

The silica NPs were added in two different types of biopolymers (xanthan gum and Scleroglucan). The effect of the recovery factor was evaluated using microfluidics tests. The capillary number was maintained low (1 × 10^−6^ and 2 × 10^−6^) to allowed the positive effect of the NPs in the reduction of the interfacial tension and the contact angle [15,18,19]. The biopolymer Scleroglucan showed poor behavior reaching the lowest (69%) and more delayed (3.8 PVI) recovery. When the NPs were added to the SCL solution, some injectivity problems were present. As a consequence, the positive results showed in the IFT and contact angle were not evaluate in microfluidic tests. In the case of the brine, the NPs improve the performance of the water flooding, increasing the recovery factor from the OOIP in 7.5% and anticipating the production reaching the steady-state 1.3 PVI early (Figure 9). The biopolymer xanthan gum showed the best performance when the NPs was added. The improvement in the recovery factor was 11.7% from the OOIP (Figure 9). In terms of time, the NPs did not change the time for reaching the steady-state. In the studies development by Maghzi et al., 2011; Maghzi et al., 2014; Cheraghian and Khalilinezhad, 2015 [20,21,22], similar behaviors were observed in the evaluation of polymer flooding with NPs.

### 4.3. NPs Effect in the Injectivity

The injectivity index was used to study the relationship between differential pressure and flow rate. The occurrence of loss of injectivity could indicate a reduction in the permeability of the porous media. In the first part of this study, the Xanthan Gum and Scleroglucan followed a similar behavior during the flooding process in comparison with the brine flooding. Still, the injectivity for both biopolymers solutions was lower than that for brine. The biopolymer adsorption could be the cause of this reduction.

In the case of the solution of Xanthan Gum with NPs, the injectivity was improved during the first 1.5 PVI. That could be due to the nanosize distribution, since a more homogeneous distribution of the NPs takes place when the biopolymer is in the solution. As a consequence, the combination of factors like interaction polymer–NPs and a low rate are controlling the polymer adsorption and NPs agglomeration, resulting in a decreased differential pressure and improved injectivity.

In a high rate scenario, the result suggested Xanthan Gum’s physical adsorption on the surface of the porous media. As a consequence, permeability is reduced and the differential pressure increases. However, after some time, the agglomeration of the NPs in the fluids without polymers affects the injectivity index and showed higher values for the biopolymer system at the end of the flooding process.

### 4.4. Effect of the Flow Rate in the Recovery Agent Performance

**Figure 10:**
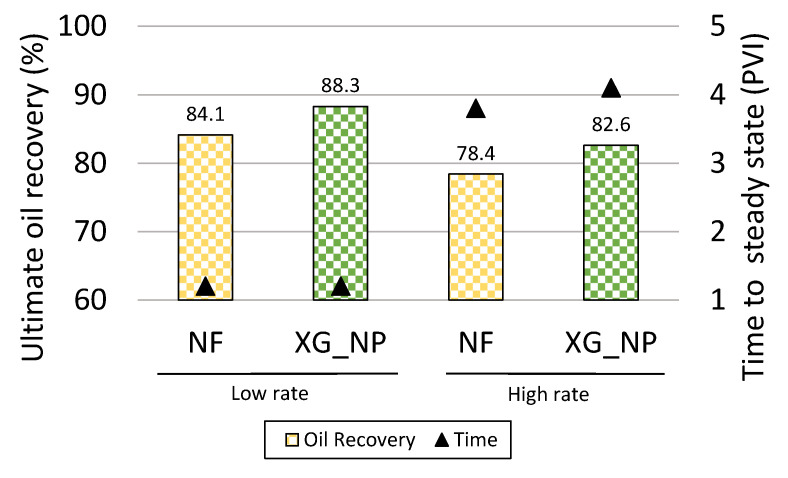
Ultimate oil recovery factor for the xanthan gum and NF at a low and high injections rate as a function of the time to reach the steady-state.

The results for the ultimate oil recovery in the reference nanofluid (NF) and the xanthan gum with NPs (XG_NP) in two flow rate scenarios were shown in Figure 10. The time (PVI) required to reach this recovery was also shown. For both solutions, the ultimate oil recovery factor was lower at a high rate in comparison with the results at a low rate (2 × 10^−6^ capillary number). Nevertheless, the high flow rate (0.39 µL/min) affected in the same proportion the performance of both solutions. The reduction in the ultimate oil recovery was 5.7% from the OOIP for the NF and XG_NP. In terms of time, at a high rate, the steady-state point was approximately three times more delayed. As a result, the improved performance of the NPs, when the xanthan gum was in the solution, was evident even in an adverse flow regime for the NPs. The results align with the observation made by Hendraningrat et al., 2013; Zhang et al., 2016 [13,29] which explains the negative impact of high flow rates in the performance of the NPs on the oil displacement.

## 5. Conclusions

This study was proposed to investigate the effect of adding silica NPs in two commercial biopolymers solutions. IFT, contact angle and nanosize distribution were measured to show the effect on the fluid-fluid and fluid-solid interactions. Moreover, the solutions were tested in water-wet micromodels to investigate the effect on the sweep efficiency and displacement efficiencies in terms of oil recovery factor, and injectivity, in addition to the effect of flow rate on the recovery. Based on the results obtained, the following conclusions can be drawn:

- The interaction between the NPs and the polymer had a minor effect on the viscosity of the polymeric solution and of the brine. When the NPs were added to the Scleroglucan, a slight increase in viscosity was reached. In the case of Xanthan Gum, the interaction with the silica NPs produced a very small decrease in viscosity.
- The Xanthan Gum and Scleroglucan did not affect the reduction in the interfacial tension and contact angle of NPs solutions. Additionally, the biopolymers prevented the agglomeration of the NPs in the solution.
- The Xanthan Gum flood led to faster and higher ultimate oil recovery, smaller remaining oil clusters, and less non-wetting phase connectivity in comparison with a conventional water flooding. However, the NPs-assisted Xanthan flooding achieved the highest ultimate oil recovery and the smallest oil clusters. Additionally, at a higher rate, the system Xanthan Gum/NP showed better performance than the NF.
- In the scenario where the Xanthan Gum and the NF systems were tested at 2 × 10^−6^ capillary number, the injectivity improved, due to a greater homogenous dispersion of the NP in the solution and the reduction in the polymer adsorption. In the case of a higher rate, the flow of the system Xanthan Gum/NP improved the injectivity after 4 PVI.

Overall, the Xanthan gum performance improved when the NPs were in the solution in comparison with the reference fluid. These results suggested an improvement in the sweep and displacement efficiencies at the same time. Opposite, the behavior of the Scleroglucan was poor in comparison with the brine and unsuccessful in the mix with NPs. In general, this work shows new research alternatives to improve oil recovery using fluid characterization and micromodel approaches.

## Figures and Tables

**Table 1:** Properties of the stock liquid surface-modified silica NPs solution.

Properties	Value
NPs concentration	26 wt.%
Basis	SiO_2_ (sol-gel-anionic)
Modification	Polymer
Average Size	32 nm
Solvent	Deionized water
Specific surface area	140–220 m^2^/g

**Table 2:** Solutions composition.

Solution Type	Sample Name	Polymer Concentration (wt.%)	NPs Concentration (wt.%)	Brine Concentration (wt.%)
Brine	Brine	0	0	3.00
Nanoparticle	NF	0	0.100	3.00
Xanthan Gum	XG	0.016	0	3.00
Scleroglucan	SCL	0.025	0	3.00
Xanthan Gum/NP	XG_NP	0.016	0.100	3.00
Scleroglucan/NP	SCL_NP	0.025	0.100	3.00

**Table 3:** Density, viscosity, pH and nano-size distribution results of the fluids used in this study.

Fluid	Density (g/cm^3^)	Viscosity (mPa·s)	pH	Nano-size Distribution
Brine	1.019	1.04	6.52	-
NF	1.021	1.07	6.72	64.82
XG	1.020	2.26	6.00	-
SCL	1.020	2.44	6.84	-
XG_NP	1.021	2.20	6.60	31.87
SCL_NP	1.021	2.46	7.22	31.48
Dead_Oil	0.892	21.9	-	-

**Table 4:** Biopolymer and NPs solutions flow rate selection at 1 × 10^−6^ capillary number.

Test No.	Recovery Agent	IFT (mN/m)	Flow Rate (µL/min)	Capillary Number	S_wi_	Ultimate Oil Recovery (frac. of OOIP)
1	Brine	18.58	0.121	1 × 10^−6^	0.13	0.76
2	XG	19.38	0.058	1 × 10^−6^	0.16	0.77
3	SCL	16.00	0.044	1 × 10^−6^	0.20	0.69

**Table 5:** Biopolymer and NF flow rate selection at 2 × 10^−6^ capillary number.

Test No.	Recovery Agent	IFT (mN/m)	Flow Rate (µL/min)	Capillary Number	S_wi_	Ultimate Oil Recovery
4	NF	6.19	0.078	2 × 10^−6^	0.17	0.84
5	XG_NP	5.92	0.039	2 × 10^−6^	0.14	0.88

**Table 6:** Biopolymer and NF flow rate selection of 0.39 µL/min.

Test No.	Recovery Agent	IFT (mN/m)	Flow Rate (µL/min)	Capillary Number	Swi	Ultimate Oil Recovery
6	NF	6.19	0.39	1 × 10^−5^	0.18	0.78
7	XG_NP	5.92	0.39	2 × 10^−5^	0.14	0.85

**Table 7:** Effect of the NPs in the performance of the biopolymer solutions at room temperature.

Variable	Reference			Xanthan Gum			Scleroglucan		
	NF	Brine	Δ	XG_NP	XG	Δ	SCL_NP	SCL	Δ
Interfacial tension (mN/m)	6.2	18.6	12.4	5.9	19.4	13.5	5.9	16.0	10.1
Contact angle (degrees)	41.9	65.3	23.4	38.4	51.6	13.2	42.3	56.0	13.7
Viscosity (mPa/s)	1.1	1.0	0.1	2.2	2.3	0.1	2.5	2.4	0.1
pH	6.7	6.5	0.2	6.6	6.0	0.6	7.2	6.8	0.4
Nanosize distribution (nm)	64.8	-	**-**	31.9	-	**-**	31.5	-	**-**

## Abbreviations

°API	Oil relative density
CNC	Critical nanoparticles concentration
D	Darcy
EOR	Enhanced Oil Recovery
g	Gravitational constant
HPAM	Hydrolyzed polyacrylamide
IFT	Interfacial tension
*I_W_*	Water injectivity
*I_index_*	Injectivity index
*I_polymer_*	Polymer injectivity
*I_reference_*	Reference fluid injectivity
Nc	Capillary number
NF	Reference nanofluid
NPs	Nanoparticles
OOIP	Original oil in place
PAM	Polyacrylamide
PVI	Pore volume injected
*q_w_*	Water rate
Ro	Radius of drop curvature
SARA	Saturate, aromatic, resin and asphaltenes
SCL	Scleroglucan
Sor	Residual oil saturation
Swi	Initial water saturation
*V_w_*	Interstitial velocity
XG	Xanthan Gum
ΔP	Pressure difference
Δ*ρ*	Density difference
*σ_ow_*	Interfacial tension
*µ_w_*	Water phase viscosity
*β*	Shape factor

## Appendix A. Segmented Images from Microfluidic Tests

Binary images after the segmentation process from the images of the microfluidic tests. The images were used the calculate the behavior of the oleic phase (white) during the time for each recovery agent in the three different scenarios of the study.

**Table A1:** Images segmented using the Matlab code in the function of the porous volume injected (PVI) during the recovery agent injection. The fluids are the brine, xanthan gum, and Scleroglucan for the microfluidic screening of biopolymer solutions.

PVI	Brine	XG	SCL
0.00			
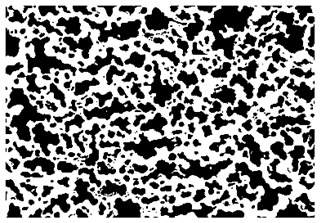
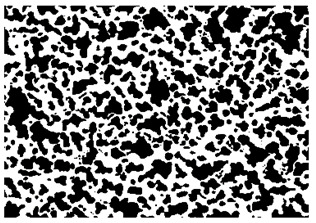
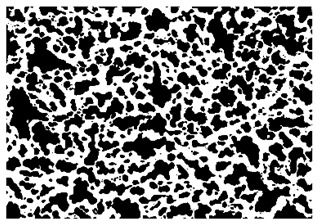
0.50			
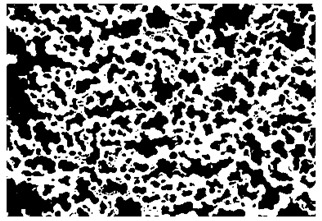
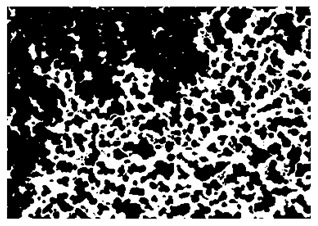
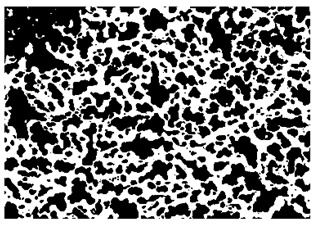
1.00			
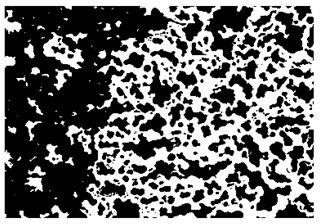
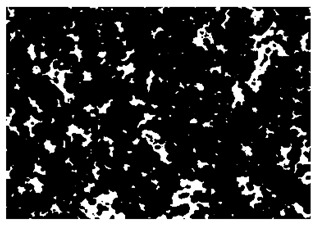
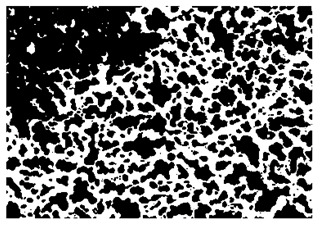
2.00			
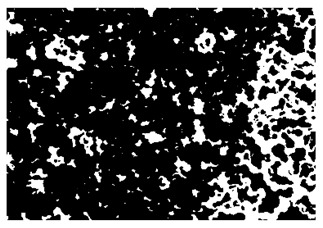
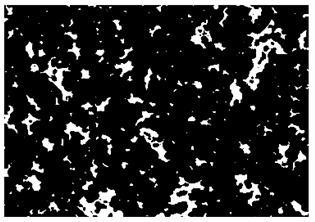
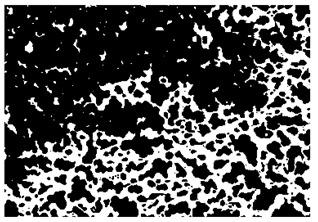
4.00			
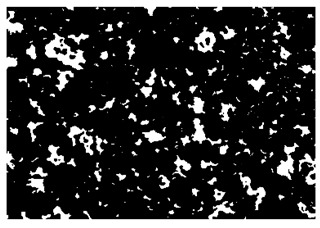
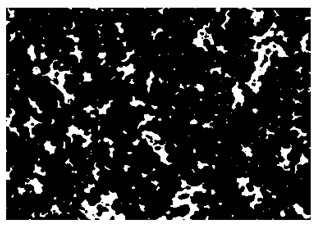
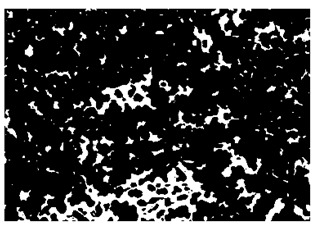

**Table A2:** Images segmented of the evaluation of the silica NPs in the polymer flooding in the function of the porous volume injected (PVI) during the recovery agent injection. This part shows the reference nanofluid (NF) and the xanthan gum with NPs (XG_NP). Due to different injection problems, the Scleroglucan with NPs was unsuccessful.

VPI	NF	XG_NP
0.00		
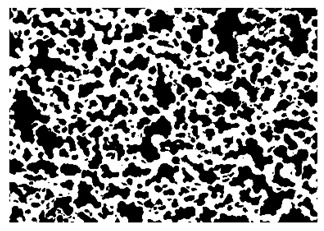
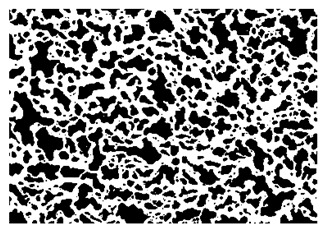
0.50		
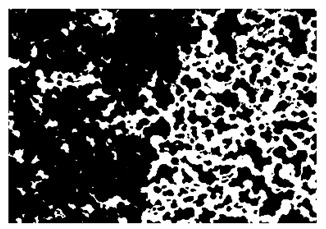
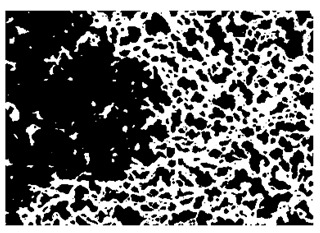
1.00		
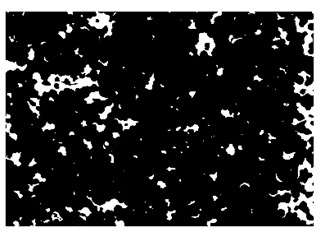
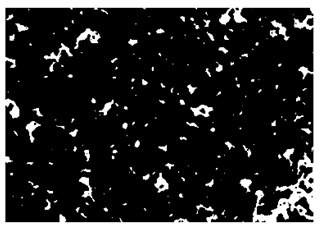
1.50		
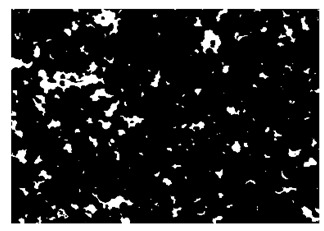
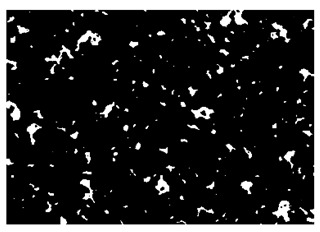

**Table A3:** Images segmented from the microfluidic tests to evaluate the effect of the flow rate on the recovery performance of the xanthan gum with NPs (XG_NP) in comparison with the reference nanofluid (NF).

VPI	NF	XG_NP
0.00		
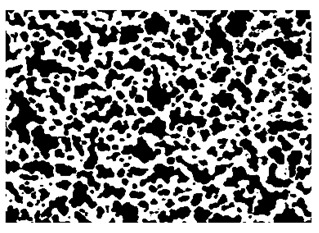
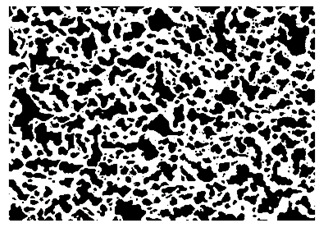
0.50		
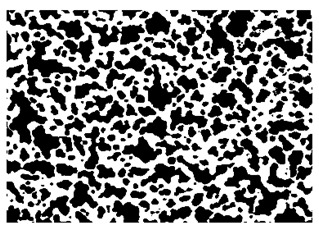
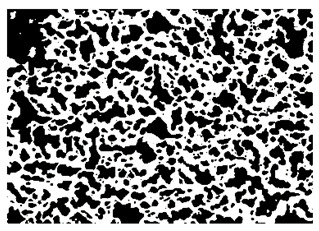
1.00		
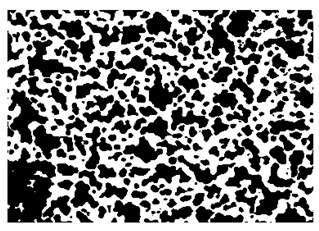
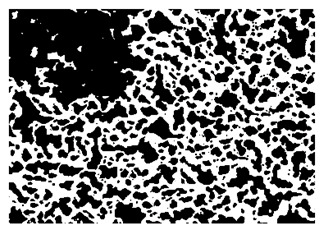
2.00		
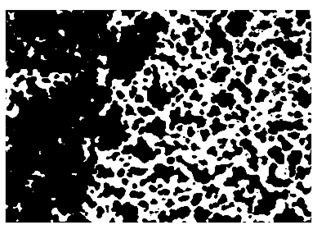
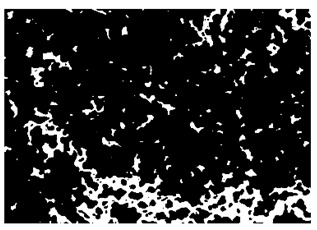
4.00		
